# Meta-analysis of generalization reliability of the Montreal Cognitive Assessment (MoCA) questionnaire in cognitive impairment

**DOI:** 10.1192/j.eurpsy.2024.1327

**Published:** 2024-08-27

**Authors:** R. Fernández Fernández, Á. Izquierdo de la Puente, P. del Sol Calderón, M. Vizcaíno da Silva, A. Rodríguez Rodríguez

**Affiliations:** ^1^Psychiatry, Hospital Universitario Infanta Cristina, Parla; ^2^Psychiatry, Hospital Universitario Puerta de Hierro Majadahonda, Majadahonda; ^3^Psychiatry, Hospital HM Puerta del Sur, Móstoles, Spain

## Abstract

**Introduction:**

Dementia is a syndrome of high prevalence and health impact. The Montreal Cognitive Assessment (MoCA) questionnaire is a screening tool whose use has increased in recent years, especially in cases of mild cognitive impairment. Some studies suggest that its ability to detect cognitive impairment, especially in early or mild stages, seems to be greater than gold-standard instruments (Ciesielska et al., 2016).

**Objectives:**

We have performed a meta-analysis of reliability generalization to see if different adaptations and use in different contexts show consistent results.

**Methods:**

We performed a literature search in PyscINFO and Medline with the terms “Cognitive impairment” AND “internal consistency” AND “Cronbach”, using the following inclusion criteria:

1. Be a study in which the MoCA scale was applied to a population sample.
2. Studies published in the last 10 years.
3. Studies that provide the reliability coefficient or sufficient data to calculate them.
4. Be written in English or Spanish.

We have limited our study to the last 10 years and the English language has given us a total of 19 results in Medline and 132 results in PsycINFO. Subsequently, we completed this search by snowball sampling.

A random effects model was assumed for the statistical calculations and the transformation of our values using the Hakstian and Whalen (1976) proposal. Statistical analysis was performed with the MAJOR package of the Janovi program, based on the R environment.

**Results:**

We obtained a mean reliability for the transformed test scores of 0.42 (95% CI: 0.38 - 0.45), as well as high heterogeneity measured by Cochran’s Q statistic and the I^2^ index, which is attributed after analysis of moderating variables to the geographical adaptation of the questionnaire and the type of patient on whom it is applied. Our Funnel Plot graph indicates that we do not appear to have committed a publication bias.

**Conclusions:**

Our meta-analysis shows high heterogeneity, mainly explained by the population of origin, both geographically (continent) and clinically (presence of primary cognitive impairment or not), with special incidence in those with impairment secondary to other pathologies, mainly neurological. However, we should consider the high probability that we have not included important variables in our analysis that could increase the explanatory power of our model.

**Disclosure of Interest:**

None Declared

